# Modeling the Structure of RNA Molecules with Small-Angle X-Ray Scattering Data

**DOI:** 10.1371/journal.pone.0078007

**Published:** 2013-11-04

**Authors:** Michal Jan Gajda, Denise Martinez Zapien, Emiko Uchikawa, Anne-Catherine Dock-Bregeon

**Affiliations:** 1 NMR-based Structural Biology, Max Planck Institute for Biophysical Chemistry, Göttingen, Niedersachsen, Germany; 2 Hamburg Outstation, European Molecular Biology Laboratories, Hamburg, Germany; 3 Laboratoire de Biologie et Génomique Structurales, Institut de Génétique et de Biologie Moléculaire et Cellulaire, Illkirch, Bas-Rhin, France; 4 Génomique Fonctionnelle, Institut de Biologie de l’École Normale Supérieure, Paris, Île-de-France, France; University of Queensland, Australia

## Abstract

We propose a novel fragment assembly method for low-resolution modeling of RNA and show how it may be used along with small-angle X-ray solution scattering (SAXS) data to model low-resolution structures of particles having as many as 12 independent secondary structure elements. We assessed this model-building procedure by using both artificial data on a previously proposed benchmark and publicly available data. With the artificial data, SAXS-guided models show better similarity to native structures than ROSETTA decoys. The publicly available data showed that SAXS-guided models can be used to reinterpret RNA structures previously deposited in the Protein Data Bank. Our approach allows for fast and efficient building of *de novo* models of RNA using approximate secondary structures that can be readily obtained from existing bioinformatic approaches. We also offer a rigorous assessment of the resolving power of SAXS in the case of small RNA structures, along with a small multimetric benchmark of the proposed method.

## Introduction

The number of functionally important RNAs of unknown structure is growing rapidly due to recent advances in transcript identification [1], [2] and expression measurement [3].

The processes for determining the structures of a few RNA families (ribosomal RNAs, tRNAs, and riboswitches) has developed to the point where the structures can be identified rapidly; however, the structures of RNAs from other families are famously difficult to solve even with state-of-art structure determination efforts [4], [5]. A notable exception are the single-particle cryo-electron microscopy methods, but these are handicapped by the relatively small particle size of most expressed RNAs (even after exclusion of all interfering RNAs), which often falls below the limit of the method.

There is also an increasing need for in-solution confirmation of determined structures, which may be done using lower-resolution approaches [6]–[9].

We propose a computational procedure that uses small-angle X-ray solution scattering (SAXS) data to obtain low-resolution approximations of RNA structures. This process can be used as a diagnostic tool to help confirm predicted secondary structures with a higher degree of certainty than chemical footprinting approaches alone [10], [11].

We use the most popular metrics to verify our approach and compare our results obtained with SAXS data with those from other approaches to RNA modeling.

Our test targets are stem loop 3 (HP3) and stem loop 4 (HP4) of 7SK RNA, which is one of the most abundant regulatory RNA in mammals [12], [13].

### Related Work

#### RNA modelling approaches

There have been previous attempts to develop junction-based RNA structure modeling methods, for example JUMNA [14], because helical regions are believed to be mostly constrained to near-ideal conformations. Alternative approaches use motif networks inferred using local similarity of sequence and secondary structure. Of these, MC-SYM [15] uses least-squares minimization of cyclic motif networks, ASSEMBLE [16] allows for hand-picking of the most appropriate motifs using human knowledge, and RNA-MoIP [17] uses an integer programming framework in order to scale to larger RNA molecules.

There have also been several attempts to develop conventional sequential fragment assembly, which works by copying local coordinates or angles, similar to the ROSETTA protein modeling method [18], [19].

Monte Carlo simulations of reduced nucleotide-based representations guided by statistical potentials have also been used [20] (released as part of the NAST nucleic acids simulation toolkit). A computationally efficient reduced model on a triangular lattice was found to outperform pure secondary structure prediction on pseudoknots [21].

#### Modelling with aid of experimental information

Modeling calls for more experimental information, such as that acquired in SAXS experiments, because previous research has shown considerable success in elucidating the general shape of RNA structures [22] and has significantly reduced the dimensionality of the tertiary structure landscape [23].

Existing approaches can be supplemented by experimental information from SAXS by adding a final step of filtering generated models to those best fitting the experimental data, as in FAST-SAXS RNA [6], [24].

Attempts have been made to produce an approximate model of flexible RNA molecules using residual dipolar coupling (RDC) data acquired from nuclear magnetic resonance (NMR) experiments to restrain relative angles between helices [25]. This approach has achieved considerable success for molecules with a small number of flexible angles [25].

Experimental data may be used not only to drive sampling process, but also to verify that the structure is not too flexible, and indeed may correspond to a unique tertiary structure. Particularly the SAXS data can readily show signs of flexible or disordered structure on a Kratky plot [26].

#### Use of RNA secondary structure

Secondary structure is an important input for most tertiary structure prediction algorithms. Use of chemical probing methods like SHAPE [11], [27] to improve local secondary structure information is therefore believed to enhance the success rate of modeling attempts.

Starting from RNA secondary structure prediction might be expected to cause problems, because any bad pairing would be propagated to the modeled three-dimensional (3D) structure, and would possibly generate errors in the selection of 3D orientation.

However, the accuracy of secondary structure modeling is reported to be more than 73% [28], with a Matthews’ correlation coefficient of 0.8 [29].

There are two ways to mitigate secondary structure errors: one is to use a consensus secondary structure prediction, and the second is to compare models created using a range of different predictions and to draw conclusions using features of the whole ensemble.

Similar to previous methods, our method capitalizes on the accuracy of secondary structure prediction in an attempt to tackle the more difficult part of the problem, which is full 3D modeling.

## Methods

Here we describe a novel fragment assembly method RFR, which uses a sophisticated, variable-length fragment database, and insights into RNA secondary structure organization in order to significantly speed up the conformational search.

### Complexity of RNA Tertiary Structure Relative to Secondary Structure

First we propose a novel way to describe the informational complexity of an RNA structure at low resolution. This description will provide the basis for the highly efficient sampling algorithms outlined in the next section.

Given certain secondary structure information, we may compute the degree of determinacy of an RNA tertiary structure as the number of degrees of freedom.

**Figure 1:**
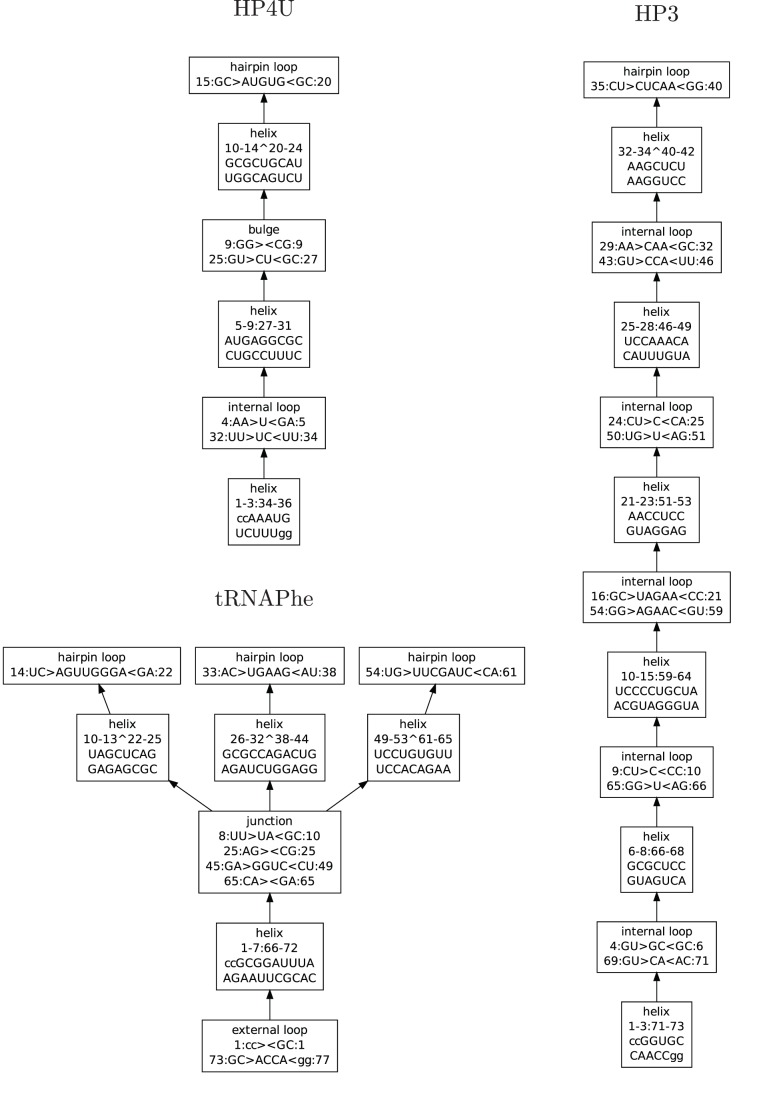
Schematic complexity of RNA secondary structures for HP4, HP3, and tRNAPhe. Secondary structure elements are represented as nodes in the graph. Each is given a descriptive name. The top nodes are always hairpin loops, and the bottom nodes correspond to the outermost helices or external loops.

We split RNA secondary structure into helices and non-helical elements: internal loops, bulges and junctions, which are treated together and called just “junctions” below. We may observe that non-pseudoknotted secondary structure forms a tree of non-helical elements joined by helices. Each subtree may be sampled separately, and most of the conformational freedom corresponds to non-helical elements. Although information fully describing RNA 3D structure may be captured by computing flexible torsion angles(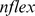 ) at high resolution [30], we use the number of junctions (and other non-helical elements) as the number of degrees of freedom to describe the complexity of the structure at low resolution. (Example decomposition of an RNA secondary structure into helices, loops, and junctions that form separate elements is shown on the Figure 1). Both types of information are sufficient to fully resolve a 3D model at low resolution, when it is does not contain long unpaired strands. Using junctions significantly reduces search space, which is explicitly constrained by helices on the ends of the junction.

The 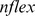 measure is used in high-resolution molecular dynamics modeling in either Cartesian or torsion angle space, whereas our method uses large-fragment replacement that can replace many tens of flexible bonds within one step. A fragment database may occasionally lack coverage; if it does, we resort to alternative strategies for subdividing secondary structure elements into strands and perform fine sampling of these subdivided flexible parts. Because these parts represent minor portions of the considered structures, we still substantially reduce the number of necessary sampling steps, while still sampling the difficult parts for which our database may lack coverage. [31] Theoretical analysis of the information content of a single SAXS experiment performed on early-generation beamlines has suggested that there are no less than 17 to 20 degrees of freedom within acquired data [32], thus justifying the hypothesis that judicious use of SAXS information alone may be used to validate the orientation of helices in structures with no less than 8–10 junctions.

### RNA Database

We started our study with the RNAJunction database [33], which contains only junction structures, and idealized helices produced by X3DNA [34]. This is not unlike the approach in [35], where RDC data were used to determine the overall orientation of the helices to create a coarse-grained model of the molecule. The results of the modeling were much more accurate for the full database extracted from the Protein Data Bank (PDB) containing the conformations of both helices and junctions (data not shown). We enriched this database with information about junctions from RNAJunction database, in order to mitigate omissions in our extraction procedure(see [33] for an analysis of the completeness of the RNAJunction database). To further ensure a complete database, we also added fragments from the LIR database [36], [37] that our procedure had not included.

Our database contains tertiary structures of a total of 33,000 secondary structure elements and more than 62,000 strands, among which there are more than 11,000 loop strands.

### Sampling Algorithm

**Figure 2:**
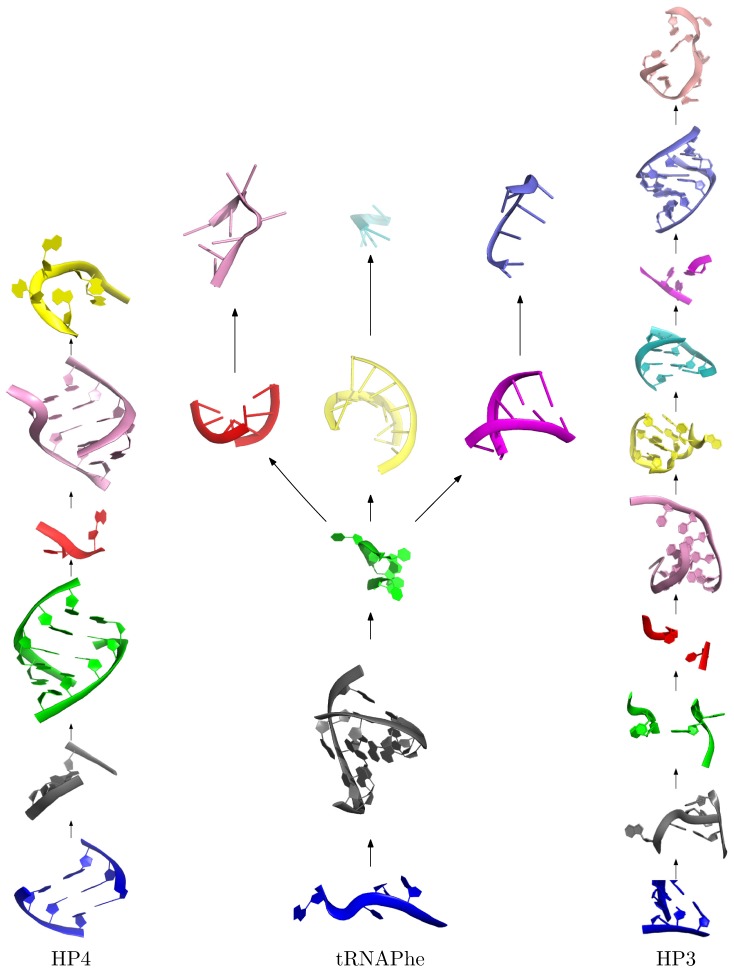
Tertiary structure elements broken down by topology. Nodes of this graph correspond directly to the nodes within the secondary structure graph presented in the Figure 1, so that three-dimensional element covers a single secondary structure element.

Secondary structure definition provided as an input, is used to split predicted 3D structure into a tree of separate secondary structure elements. Thus, replacement of an element in any point of the tree with a 3D structure would only affect the placement of elements below it in the tree hierarchy (see Figures 1, and 2).

For each of the secondary structure elements, we perform a database search to find fragments matching the number and lengths of the strands. These fragments are then scored in terms of sequence similarity (see below).

In the rare event when there are not enough fragments found, we mark this element for sampling using variant B of the algorithm, and search the database by strands for each strand within the element.

Then we loop the annealing protocol through about 20 stages of decreasing temperature (to 80% of the previous value after each stage), making 100 fragment exchange attempts within each stage. At each sampling step, the algorithm replaces the structure of a single 3D element (matching a single element of secondary structure). Most elements are subject to whole-fragment replacement and then scoring (as explained in the next section), after which replacement is accepted with a probability 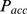 corresponding to a modified Metropolis-Hastings Monte-Carlo criterion [38].
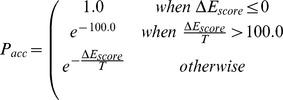



Where 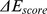 is the difference in scores between the old and new model (in that order, see details of the scoring function in the next section). Temperature 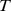 is expressed in arbitrary units to which weights of the scoring function are calibrated. The starting temperature 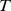 is computed as 20% of the initial scoring function value, or 1.0, whichever is greater, in order to ensure that sampling can easily jump over reasonable local minima, given that the initial randomly drawn structure is supposed to represent a bad fit.

Each replacement step consists of superimposing boundary atoms onto the boundary atoms of the previous element in the topology of the secondary structures, and then minimizing the 3 last dihedral angles of those boundary elements to assure a contiguous backbone.

In the case of insufficient fragment coverage, version B of the sampling scheme replaces all strands within an element instead of replacing fragments. After such a replacement, an additional term that corresponds to the consistency of the different strands (contiguity) is added to the score.

The main contribution to the scoring function 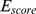 is the fit to the SAXS data valued as 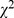 fit. Additional components just increase bias towards the high-resolution integrity of the structure, while still permitting some variation in physically plausible geometry, in case the fragment coverage does not suffice for accurate modeling. Experimental data are acquired by SAXS experiments and then fitted by scaling intensity so that the best 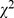 measure is obtained using the CRYSOL program [39].
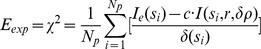



Where 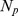 is the number of points of experimental data; 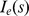 is the experimental data curve; 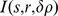, a theoretical curve parameterized by 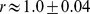, is the volume displaced by each atomic group; and 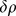 is the average excess electron density of the solvation shell layer.

This algorithm benefits from relatively few degrees of freedom and thus enjoys faster convergence than a conventional fixed-fragment-size algorithm like ROSETTA for RNA [40].

### Scoring Function

The scoring function is a sum of heuristic terms corresponding to the similarity of database fragment sequences to modeled sequences, geometric model quality terms, and fit to experimental data:
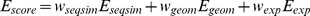




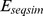
 is a fragment match score that takes into account consistency of the sequence between the fragment and a modeled sequence.


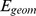
 is a geometric quality score that is a weighted sum of the contiguity score 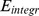 (measured as a sum of excess bond lengths in the covalently bound backbone) and a steric clash count 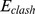 (measured as a count of clashing atoms, and then multiplying clashes between phosphorus and backbone by 5 to increase their contribution).


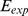
 is fit to experimental small-angle scattering data.


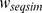
, 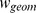, and 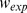 are weights optimized by regression and corresponding to the given energy functions.

The first round of benchmark simulations were computed with only 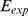 and with 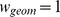, all other weights being set to 0. Regression analysis was then performed by using Waikato Environment for Knowledge Analysis [41] and selecting weights corresponding to the best match of the linear combination to the Fidelity Index value (see the next section).

### Measurements of Prediction Success

We measured prediction success using both pre-established methods (the Interaction Network Fidelity index 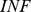, the root mean square deviation 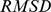 and the global distance test 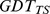) and adaptations of these methods that we believe are more applicable to RNA structures. We propose a global distance test with RNA-adapted thresholds 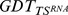, and the Fidelity Index 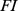, which uses a combination of 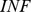 and 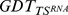 to compute a score from 0.0 to 1.0, where 1.0 corresponds to perfect accuracy.

*RMSD Root mean square deviation on single backbone atom* is standard superposition quality measured on all atoms of a high-resolution structure determined by macromolecular crystallography, on backbone atoms for NMR structures and high-resolution models, or just on a single “reference” backbone atom for big or low-resolution models.
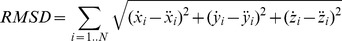



*INF Interaction network fidelity* [15] is a Matthews’ correlation coefficient for a hydrogen-bonding network between a reference structure and a model.
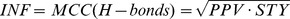


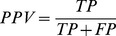


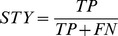



Where:

*TP* is the true positive rate (number of correctly predicted hydrogen bonds),

*FP* is the false positive rate (number of hydrogen bonds that occur only in the model structure),

*FN* is the false negative rate (number of hydrogen bonds that occur only in the reference structure).

*STY* sensitivity of the prediction, measured as a ratio of correctly detected bonds among all bonds in the native structure.

*PPV* specificity of the prediction, measured as a ratio of correct bonds among all bonds in the predicted model.

*DI Deformation index* is a compound measure of 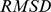, and 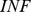 that was suggested by [15] as a more sensitive quality measure than its component measures alone:
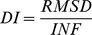



*GTD_TS_^RNA^ Global Distance Test modified for RNA*, with adapted thresholds of 1.5, 3.0, 6.0, and 12 (instead of the 1, 2, 4, 8 Å used in protein comparisons, due to the larger average distance between phosphorus atoms than between C-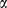 atoms) is computed on backbone phosphorus atoms instead of C-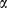. To compute this score, we patched the TMscore program used for computing protein structure similarity scores for protein models [42] (see patch in File S3).

*GTD_TS_ Global Distance Test* on backbone phosphorus atoms, with traditional thresholds (see patch in File S2).

*FI Fidelity Index* is a composite score based on the adapted 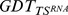 and 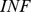 and scaled to deliver 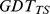 -like range of values in the range of 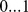.
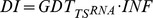



−lg _10_ *P(RMSD)* is a estimation of likelihood to built a model of given accuracy given by [?] for simple RNA with known secondary structure. Where:
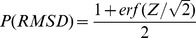


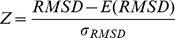


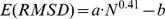


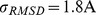


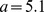


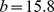



*P(RMSD)* is probability to build a model for simple RNA with given number of bases.

*E(RMSD)* is expected average RMSd for models built by molecular dynamics approach of [10].

*Z* is Z-score between a given 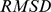 and 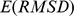.

*a, b,* 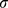 *_RMSD_* are parameters estimated by regression in [10].

*N* is a length of a modeled RNA in bases.

### Sample Preparation

**Figure 3:**
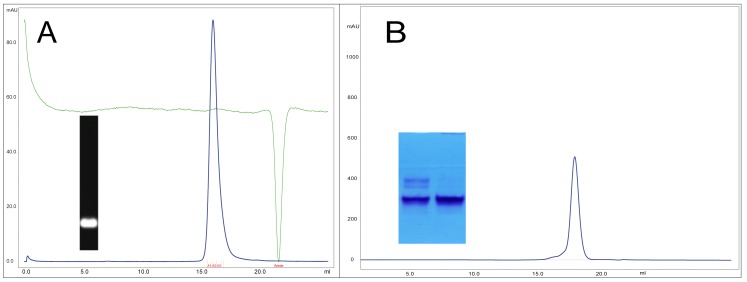
RNA sample quality controls. Control of RNA samples with size exclusion chromatography in Superose 6 (buffer Hepes pH 7.2, 20 mM, KCl 100 mM). Absorption at 254 nm (blue curve) and conductivity (green curve) are shown. A. **HP3**. Inset: Electrophoresis in native conditions (agarose 2%, GelRed coloring) of the HP3 sample measured. B. **HP4**. Inset: Electrophoresis in native conditions (acrylamide 15%, toluidine coloring) of the HP4 sample before (left) and after (right) thermal treatment (3 min 85°C).

RNA was transcribed at preparative scale (5 ml) from a linearized pHDV template [43]. This template introduces a 3′ co-transcribed HDV ribozyme, which allows cleavage in the presence of 40 mM MgCl_2_, thus ensuring a well-defined 3′-end. Preparative gel purification on acryl-urea gels allowed the ribozyme and uncleaved transcript to be removed. The purified RNA was eluted from the gel, filtered through glass wool, and then further purified on a monoQ column in Bis-Tris 20 mM pH 7.0 and a NaCl gradient from 0.1M to 1M. The fractions containing the RNA of interest were pooled and concentrated (Amicon Ultra-4), then dialyzed into “SAXS buffer” (10 mM sodium cacodylate buffer, pH 6.5, 6 mM MgCl_2_, 0.25 mM EDTA, and 100 mM NaCl) for at least 15 hours in Gebaflex-mini dialysis tubes. In the course of our functional studies involving HP3 and HP4 from 7SK, we checked that for RNA of that small size, this process leads generally to monodisperse preparation with one single conformation. A size-exclusion chromatorgraphy of a sample HP3 shows indeed a sharp, unique peak, and a single band on a native gel (Figure 3A). For HP4, we sometimes observed a small amount of a larger species (Figure 3B). That was attributed to the duplex form of HP4, since larger molecule does not appear on denaturing gel. This form was eliminated by a thermal treatment (3 min 85°C) before to SAXS measurement. Full conversion into a single conformer was monitored by native acrylamide gel (Figure 3B).

### SAXS Experiments

**Figure 4:**
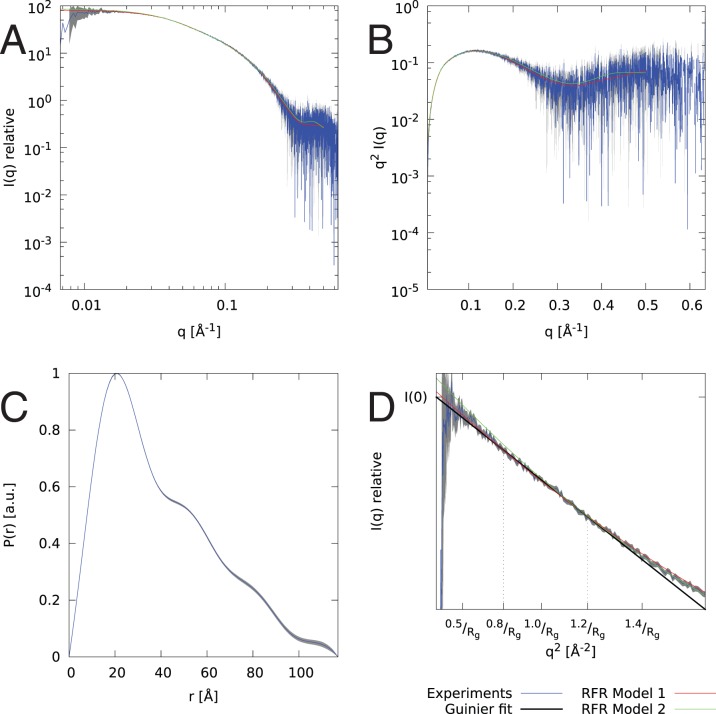
SAXS data and fit for the models of stem loop 3 (HP3). The plots are: (A) log-log data plot, (B) Kratky plot showing that RNA is compact and folded, (C) P(r) plot showing approximated distribution of interatomic distances within particle, and (D) Guinier plot with shown 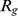, and 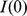 fit. Gathered experimental data is drawn in blue with gray error bars, whereas fits are drawn in red for secondary structure from [12] with 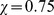, and green for secondary structure from [13] with 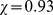.

**Figure 5:**
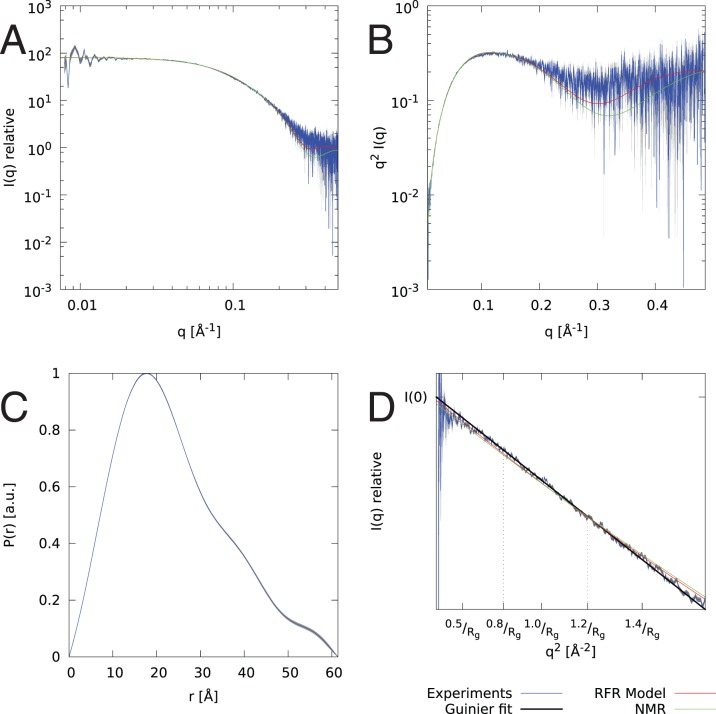
SAXS data and fit for the model of HP4. Plots of SAXS data and fit to the model (dashed red line) of stem loop 4 (HP4) against gathered experimental data (blue line with gray error bars), and fit to the NMR model (green line). The data is shown with log-log data plot (A), Kratky plot showing that RNA is compact and folded (B), P(r) plot showing approximated distribution of interatomic distances within particle (C), and Guinier plot with shown 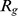, and 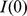 fit.

Samples at concentrations of 2.2–3.8 mg/ml for stem loop 3 of 7SK RNA (HP3) and 0.7 mg/ml to 1.2 mg/ml for stem loop 4 of 7SK RNA (HP4), both in 50 mM cacodylate buffer at pH 6.5, were measured on the X33 beamline [44] operated by European Molecular Biology Laboratory at DORIS III storage ring. Each sample was exposed for 8 frames of 15 seconds each to 1.5 Å X-ray wavelength at 10°C. Scattered radiation was recorded with a Pilatus 1M photon-counting detector. Data gathered for all samples was checked for presence of expanded or disordered conformations using a Kratky plot. All of these seem to be compact, monodisperse conformations as indicated in the Figure 4 and 5. Data parameters are given in table 1. The SAXS data for the concentrations used for structural modeling are included in File S4 for HP3 and File S5 for HP4.

**Table 1:** Data parameters.

Data collection parameters			*HP3*	*HP4*	
Instrument	X33 (EMBL, DORIS ring, DESY)				
Beam geometry		2 mm×0.6 mm			
Wavelength [Å]		1.5			
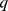 range [Å^−1^]		0.0074-0.5			
Exposure time [s]		8×15			
Temperature [K]		283			
Concentration range [mg/ml]		2.2–3.8			
		0.7–1.2			
**Structural parameters**					
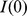 [% 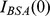 ] from P(r)		53±5		32.25±12	
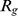					
[Å ] from P(r)		31.2±1.2		18.8±0.1	
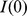 [% 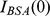 ] from Guinier		72.8±9		31.6±0.06	
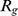					
[Å ] from Guinier		29.3±2		17.8±2.1	
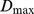 [Å] from data		117		61.4	
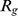					
[Å ]	*Model 1*	30		17.4	
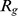					
[Å ]	*Model 2*	33			
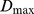 [Å] of model envelope	*Model 1*	112		61.43	
	*Model 2*	124			
Porod volume estimate [Å]		30230		10950	
Dry volume calculated from model [Å]	*Both models*	28900		14100	
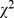 fit	Model 1	0.75		1.07	
	Model 2	0.93			
**Molecular-mass determination**					
Molecular mass 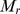 from I(0) in P(r) [kDa]		18±2		10.7±3	
Calculated monomeric 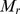 from sequence [kDa]		23.4		10.3	
Calculated monomeric 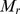 from I(0) in Guinier [kDa]		25±3		11.6	
**Software employed**					
Primary data reduction		PRIMUS			
Data processing		GNOM			
Ab initio analysis		DAMMIF			
Model comparison		SUPCOMB			
Tertiary structure modelling		RFR *(this paper)*			
Computation of model intensities		CRYSOL			
Three-dimensional graphics representations		PyMol			

### SAXS Envelope Modeling

Consistency checks between frames were performed automatically by X33 automated processing system [45] using ATSAS software [46]. The expected molecular masses of the solutes were estimated from intensity extrapolated to zero angle, and were found to be consistent with the expected masses for the monomers (see table 1. The maximum diameter of each particle was estimated by indirect Fourier transform using GNOM software [47].

*Ab initio* structures of the monomers were obtained using the program DAMMIF [48], which uses Monte Carlo simulated annealing to build the compact and contiguous bead model of uniform scattering length density that has the least discrepancy between experimental and forward-calculated scattering curves.

Calculations for HP4 were performed in the volume of a sphere with a diameter of 80.3 Å, and for HP3 in the volume of a sphere with a diameter of 109.7 Å. The calculations were visually compared between 10 different runs for each concentration to ensure consistency. We show the centroid model of the largest cluster. Average similarity between all models measured by 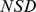 was 0.79 for HP4 and 0.70 for HP3.

### Model Visualization

Atomic model structures were superimposed over SAXS envelopes using DAMSUP software [49]. Superpositions between atomic models were then improved using PyMol [31], to minimize discrepancy between model best fitting to the envelope, and the other model.

Secondary structure trees were created using GraphViz [50] out of three dimensional fragments rendered using PyMol [31].

## Results

### Benchmark Results

We have tested our method on a set of experimentally determined RNA molecules of up to 70 nucleotides (including subset of previous benchmark [10]), as presented in table 2. SAXS input data of 200 points between 0 Å^−1^ and 0.5 Å^−1^ were backcalculated for each target by using CRYSOL [39] (data and models are included in File S6). Scattering intensity was then perturbed by adding 2% relative noise to each point, and absolute noise of 5% of the minimal intensity value.

**Table 2:** Benchmark results.

	Length in nucleotides	Number of SS elements	SAXS fit	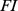	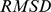	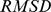 _expected by chance_	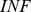	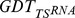	ROSETTA 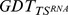 for best SAXS fit	ROSETTA average and stdev 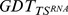		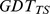	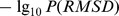	Iterations taken to find solution	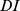
1Q9A	26	5	1.03	0.73	1.77	3.60	84%	0.88	0.70	0.49	0.80		0.81	1261	0.47
1L2X	27	3	1.15	0.14	10.81	3.90	27%	0.50	0.44	0.41	0.41		0.00	1657	0.03
2LJJ	27	4	1.15	0.33	6.62	3.90	55%	0.59	0.34	0.38	0.45		0.03	2448	0.08
1DDY	35	3	1.14	0.08	14.48	6.11	22%	0.37	0.33	0.34	0.29		0.00	1711	0.01
2L3E	35	4	0.94	0.39	5.56	6.11	61%	0.63	0.35	0.34	0.54		0.42	1064	0.11
2AU4	41	6	1.05	0.12	11.49	7.58	34%	0.37	0.29	0.21	0.30		0.01	2922	0.03
1XJR	46	9	1.11	0.11	7.87	8.71	27%	0.40	0.37	0.28	0.28		0.49	1100	0.03
3E5C	53	8	1.14	0.33	6.70	10.17	60%	0.55	0.30	0.29	0.42		1.57	2923	0.09
2KZL	55	6	1.35	0.20	9.14	10.57	49%	0.41	0.23	0.28	0.29		0.67	2899	0.05
1U8D	67	6	1.17	0.09	3.88	12.79	30%	0.32	0.19	0.18	0.25		6.43	1512	0.08

In equational form:
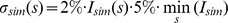


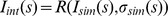



Where:

*I_sim_* is intensity simulated by CRYSOL;

*R*(*μ,* 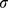) is a Gaussian random variable with median of 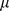, and standard deviation of 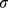;

*I_int_* is final intensity used for benchmark;


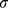
 *_sim_*(s) is apparent error produced in benchmark.


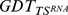
, 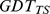, 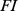, 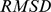, 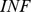, 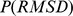, and 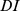 were used to estimate method success. To facilitate the assessment of convergence speed, we have added the number of iterations (out of a maximum of 3000 for all runs) needed before the minimum-energy structure was found. Length in nucleotides and the number of different secondary structure elements are also indicated to facilitate assessment of the structure’s complexity (table 2).

For better comparison we used ROSETTA software, with postfiltering by CRYSOL to improve on otherwise poor average decoy scores, which suggest that indeed producing good models is much harder than estimated by equation used to compute P-value [10]. For each target, a 100 decoys were generated, and then one with the best fit to simulated SAXS data was chosen (and included in File S6). It is worth noting that the method presented in this paper does usually achieve a slightly better result using just a single annealing run. This comparison proves that while ROSETTA computed for a longer time, and produced more decoys, it didn’t propose better models, even after filtering by fit to the SAXS data.

**Figure 6:**
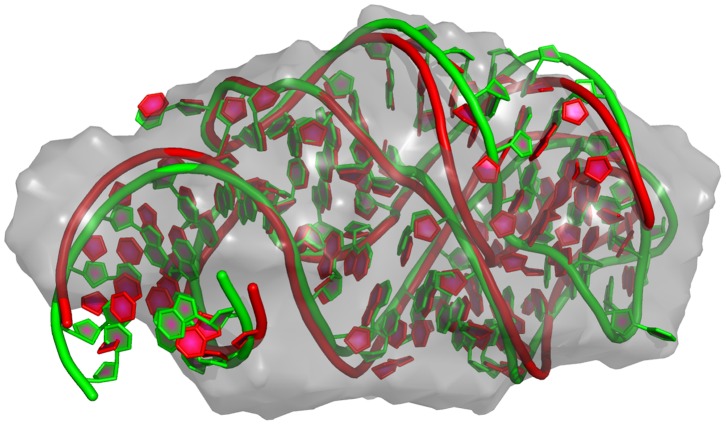
Model of 1U8D against native structure and SAXS envelope. Comparison of the red model, and green native structure for the longest modeled RNA, 1U8D. Even though shape (grey) would seem a weak restraint, topology and contacts within the model structure correspond closely to the native (similarity 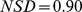 between the model to either native, or reconstructed shape).

The comparison of the accuracies and expected 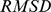 values from random modeling (table 2) clearly indicate the size and complexity of molecules that may be successfully modeled without further restraints. Although the models for the two molecules with the simplest topologies (1L2X and 1DDY) could theoretically have models of similar quality generated by chance, provided secondary structure restraints, the three other models show significant predictions, as indicated by 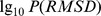 [10], in particular for the largest molecule – a guanine-responsive riboswitch with a PDB ID of 1U8D, Figure 6.

The comparison of the number of secondary structure elements and the SAXS fit seem to indicate a limitation on the maximum complexity of the modeled structure, at least when conformational space is not further restrained. As an example of built models, we show the longest modeled molecule (1U8D), where a 67-nucleotide model reached an accuracy of 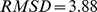 Å and 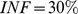 (fig. 6).

### Comparison of the HP4 Model to the NMR-derived Structure

**Figure 7:**
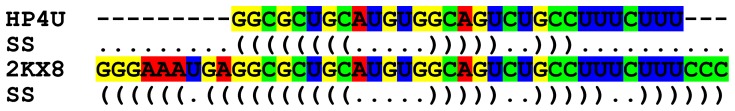
Sequence comparison for the SAXS (HP4) and NMR (2KX8) constructs. Different nucleotides have different colors to facilitate recognition of differences between the sequences. Parentheses represent nucleotide pairings, and dots represent unpaired nucleotides. Note that NMR structure has one additional outer helix to facilitate expression [51].

SAXS data were measured for stem loop 4 (HP4) of 7SK RNA. The sequence used for the SAXS analysis (shown in Figure 7) is similar to that used for the NMR analysis (deposited in PDB as 2KX8 [51]). Both are parts of the Homo sapiens 7SK sequence. We used a construct encompassing nucleotides 302–332. For the NMR study, a longer 296–331 sequence was used, with an addition of 3 G-C base-pairs forcing the structure into a hairpin. We compared the parameters inferred from the data, our model, and the NMR structure after cutting the outermost helix which was added to increase stability of the structure in [51] (see File S1 for secondary structure of HP4). Model shows not only a good 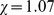 fit to data, but also a very consistent 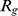 (17.8 Å from Guinier, and 17.4 Å for the model and 17.5 Å for the NMR structure without tailing helix) and 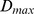 (61 Å from data and the “RFR model” and 65.7 Å for NMR structure without tail) values with those estimated from the data.

**Figure 8:**
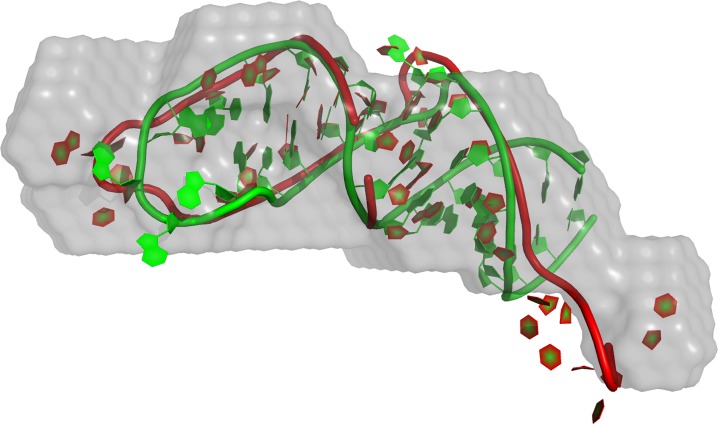
SAXS model of stem loop 4 versus the NMR structure. The gray envelope is a shape reconstruction using SAXS data, the red is a model using SAXS data, and the green is an NMR-based structure deposited in PDB. Tails of the model and the native structure that do not share the common secondary structure, are marked with shades of grey.

Due to the elongated conformation of the hairpin and the relatively small number of degrees of freedom, the model with SAXS fit of 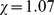 shown in Figure 8 was built with weights of 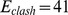, and 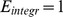. Thus, the scoring function was clearly dominated by SAXS data (see fit on Figure 5). The model was built without any fragments from the NMR structure (to validate benchmarking) and for 23 nucleotides in the same structural context are still within 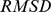 of 3.7 Å, 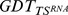 of 0.55 and 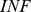 of 0.54 of the NMR structure (see model in FIle S5). Shape similarity metric 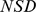 [49] between model and the reconstructed shape is 1.01.

The SAXS-based shape reconstruction is more difficult than building a model for NMR structure due to an unpaired UUUCUUU tail instead of few additional bases that allow to form the helix that is present in the NMR structure (see Figure 7). However our model still matches the bend of the main body in the NMR structure.

### HP3 Model Proposal

**Figure 9:**
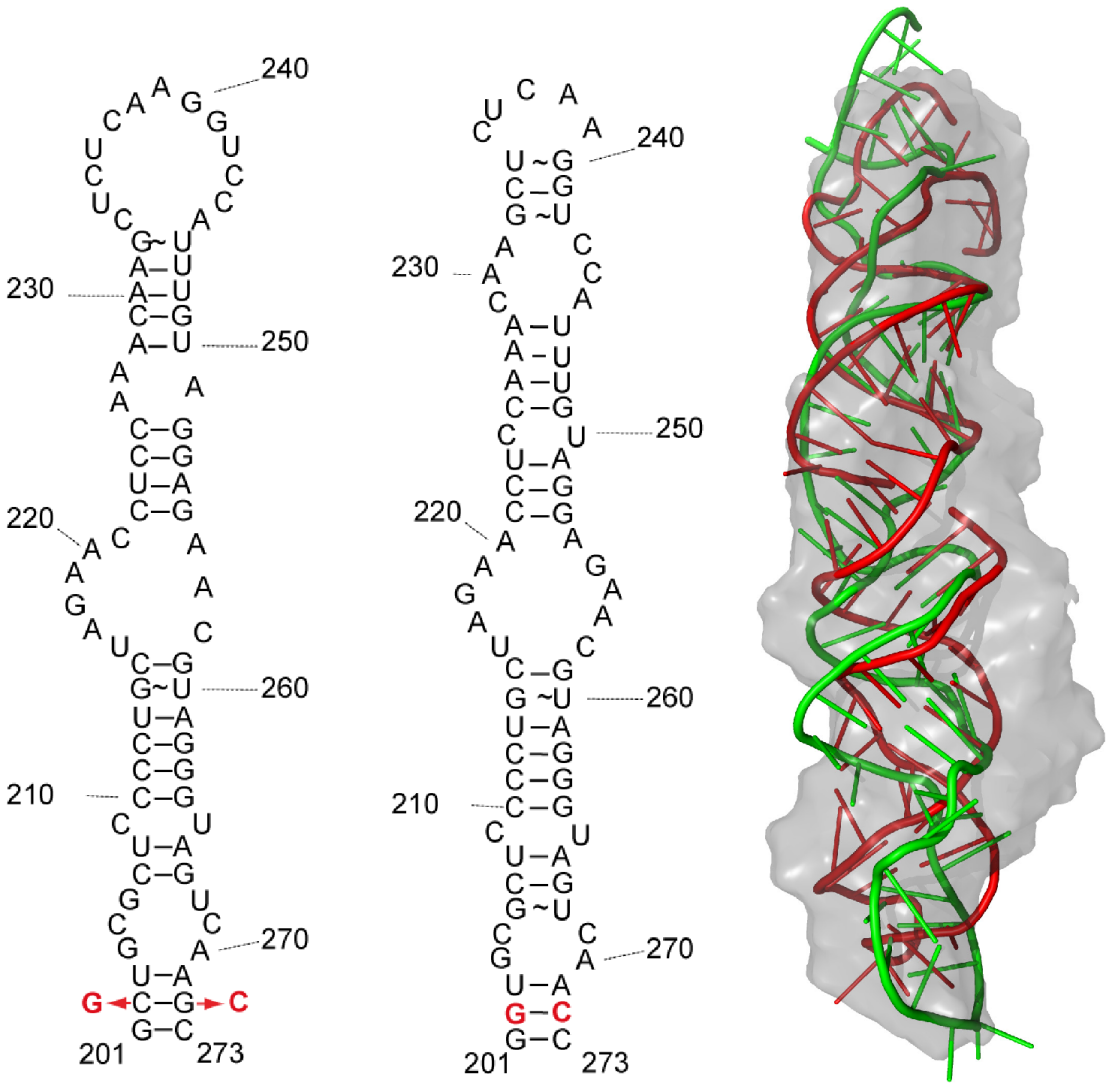
Proposed models of stem loop 3 (HP3). Models (right) depending of input secondary structure (red from [12] corresponding to secondary structure on the left, and green corresponding to secondary structure plot in the center from [13]). Nucleotides in red are changes made for facilitating the production of HP3.

We also used our method to propose two models for the stem loop 3 (HP3) of 7SK RNA (fig. 9), in accordance with two secondary structures of 7SK proposed by either [12] and [13]. The latter model [13], was based upon evolutionary analysis of 7SK which suggested a more symmetric secondary structure model than the earlier study based upon chemical probing experiments [12]. Both base-pairing schemes give similar stability. Both models of this longer element closely match SAXS data (first with 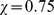, and second with 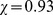, see model and data in File S4 ), and corresponding secondary structures (see model on Figure 9, and fit on Figure 4). Both models strongly suggest an extended conformation, with only slight bends in absence of protein partners. The resolution of SAXS data does not permit us to see loop details. Experimentally estimated 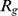 of 29 Å from Guinier or 31 Å from P(r) speaks in favor of the model built on the first secondary structure with 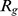 of 31 Å, instead of 33 Å as model built on second structure. Estimate of 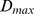 at 117 Å seems to be in between values for two models: 112 Å, and 124 Å.

Shape similarity metric 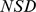 [49] between DAMMIF reconstruction and either model is 1.0.

## Discussion

We report a novel method for modeling RNA structures using pre-established secondary structure predictions, SHAPE-based determination for improved accuracy, and low-resolution tertiary structure reconstruction using SAXS data.

Used together, these methods show great potential to overcome the difficulties currently seen in determining RNA structures using crystallography and NMR.

The accuracy of the method is mostly limited by the discriminative power of available SAXS data and may be enhanced by gathering multiple data sets for components of a larger structure.

### Software Availability

Software source is available upon request from corresponding author as a Python package.

## Supporting Information

File S1 **Secondary structure of HP4.** Secondary structure diagram of HP4. Base numbering corresponds to the full human 7SK sequence.
(PNG)

File S2
**Patch for** 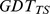. Patch modifying TMscore [42] to compute classical 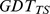 on RNA backbone.(PATCH)

File S3
**Patch for** 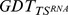. Patch modifying TMscore [42] to compute 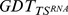.(PATCH)

File S4 **Model and SAXS data for HP3.** Archive with a model of HP3 in.pdb format, and SAXS data used to compute it in.dat format.
(ZIP)

File S5 **Model and SAXS data for HP4.** Archive with a model of HP4 in.pdb format, and SAXS data used to compute it in.dat format.
(ZIP)

File S6
**Benchmark data.** Simulated SAXS data, fit plots, RFR models, and best ROSETTA decoys (by 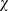) for 10 benchmark targets. Within the archive, simulated SAXS data are named in benchmark_models/*.dat, fit plots are named benchmark_models/*_fit.pdf, RFR models are named benchmark_models/*model.pdb, and top ROSETTA decoys are named rosetta_decoys/*.pdb.(ZIP)
