# Supplementary figures and images for: Modeling the Structure of RNA Molecules with Small-Angle X-Ray Scattering Data
Source: PLoS One. 2013 Nov 4;8(11):e78007. doi: 10.1371/journal.pone.0078007 (PMC3817170; doi:10.1371/journal.pone.0078007)

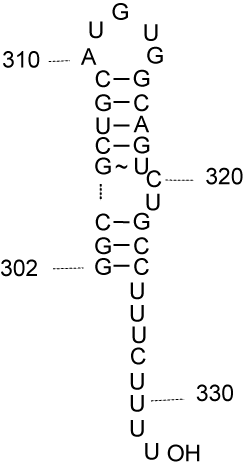

Supplement: File S1 — Secondary structure of HP4. Secondary structure diagram of HP4. Base numbering corresponds to the full human 7SK sequence. (PNG) [file pone.0078007.s001.png]

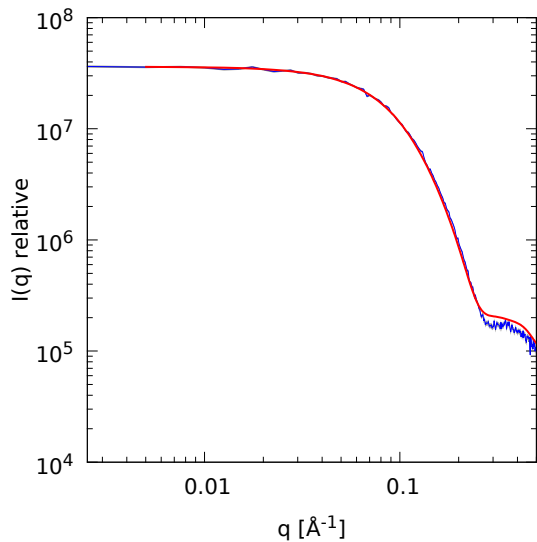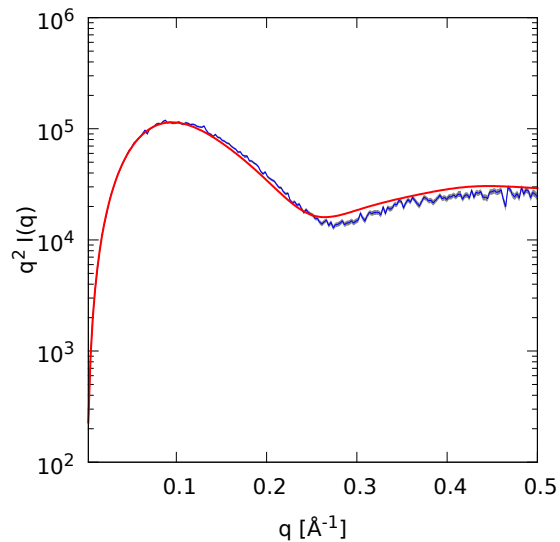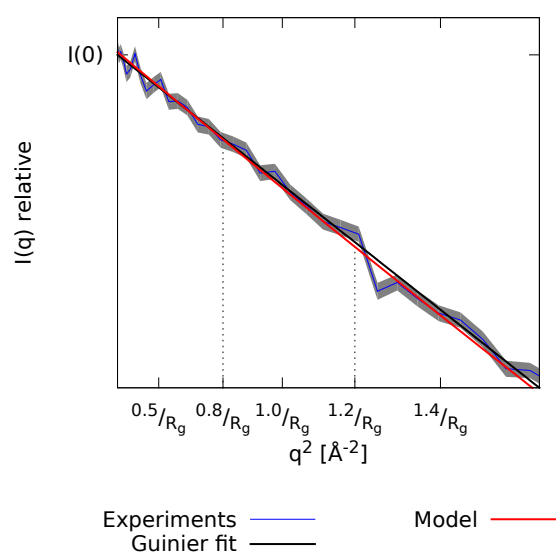

Supplement: File S6 — Benchmark data. Simulated SAXS data, fit plots, RFR models, and best ROSETTA decoys (by ) for 10 benchmark targets. Within the archive, simulated SAXS data are named in benchmark_models/*.dat, fit plots are named benchmark_models/*_fit.pdf, RFR models are named benchmark_models/*model.pdb, and top ROSETTA decoys are named rosetta_decoys/*.pdb. (ZIP) [file pone.0078007.s006.zip › benchmark_models/1U8D_fit.pdf]

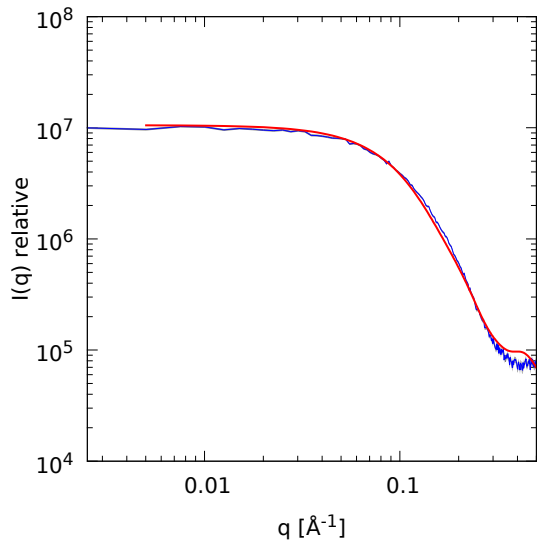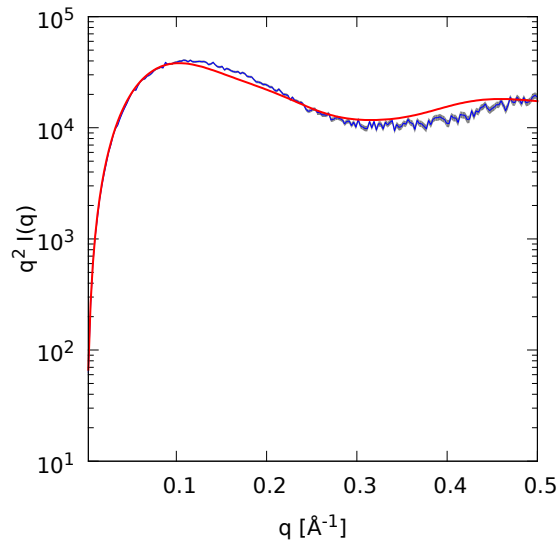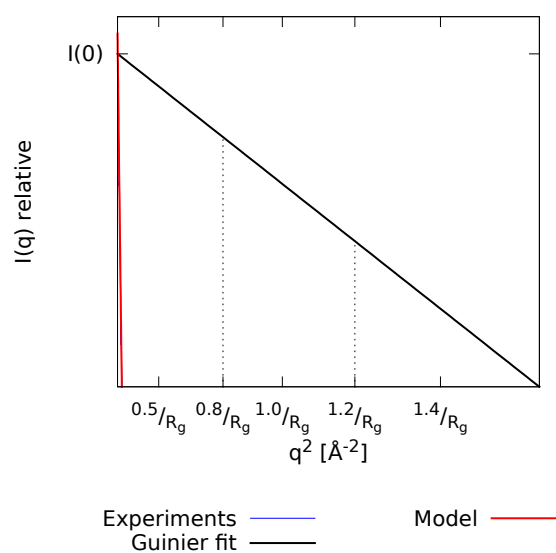

Supplement: File S6 — Benchmark data. Simulated SAXS data, fit plots, RFR models, and best ROSETTA decoys (by ) for 10 benchmark targets. Within the archive, simulated SAXS data are named in benchmark_models/*.dat, fit plots are named benchmark_models/*_fit.pdf, RFR models are named benchmark_models/*model.pdb, and top ROSETTA decoys are named rosetta_decoys/*.pdb. (ZIP) [file pone.0078007.s006.zip › benchmark_models/2AU4_fit.pdf]

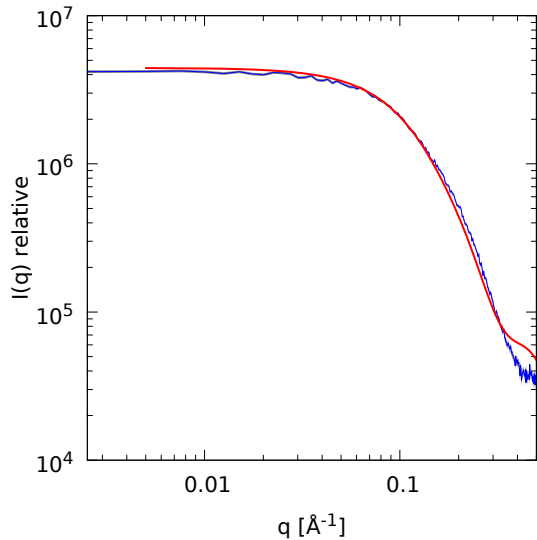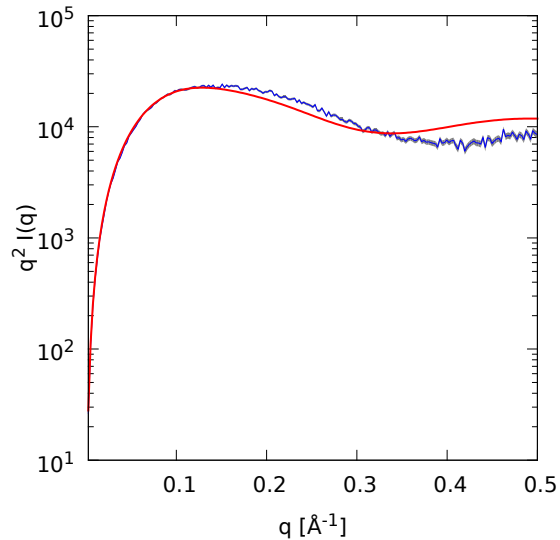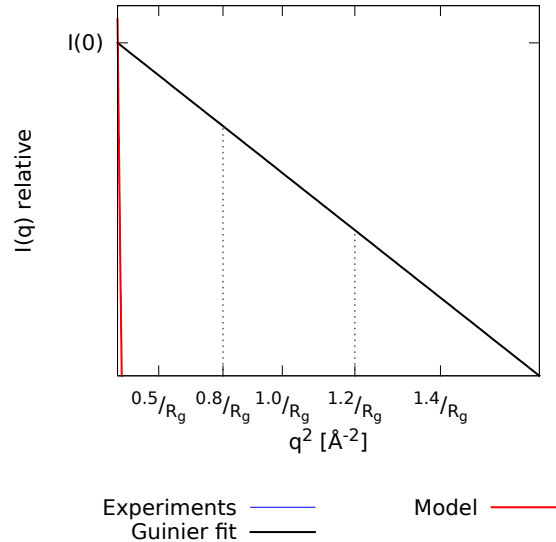

Supplement: File S6 — Benchmark data. Simulated SAXS data, fit plots, RFR models, and best ROSETTA decoys (by ) for 10 benchmark targets. Within the archive, simulated SAXS data are named in benchmark_models/*.dat, fit plots are named benchmark_models/*_fit.pdf, RFR models are named benchmark_models/*model.pdb, and top ROSETTA decoys are named rosetta_decoys/*.pdb. (ZIP) [file pone.0078007.s006.zip › benchmark_models/2LJJ_fit.pdf]

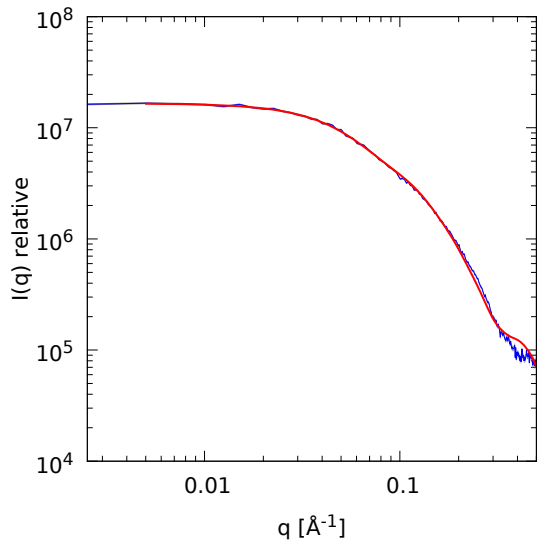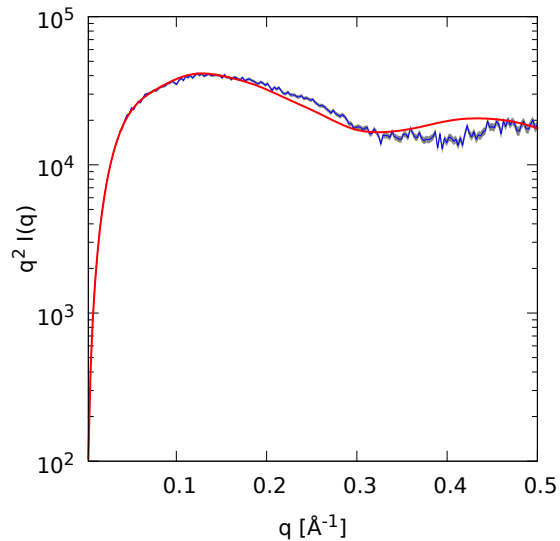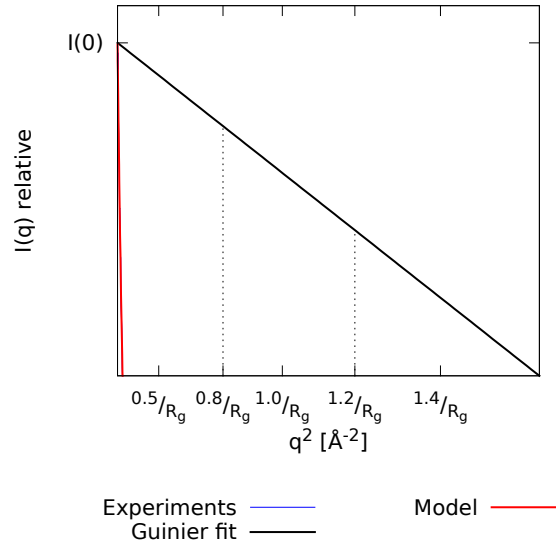

Supplement: File S6 — Benchmark data. Simulated SAXS data, fit plots, RFR models, and best ROSETTA decoys (by ) for 10 benchmark targets. Within the archive, simulated SAXS data are named in benchmark_models/*.dat, fit plots are named benchmark_models/*_fit.pdf, RFR models are named benchmark_models/*model.pdb, and top ROSETTA decoys are named rosetta_decoys/*.pdb. (ZIP) [file pone.0078007.s006.zip › benchmark_models/2KZL_fit.pdf]

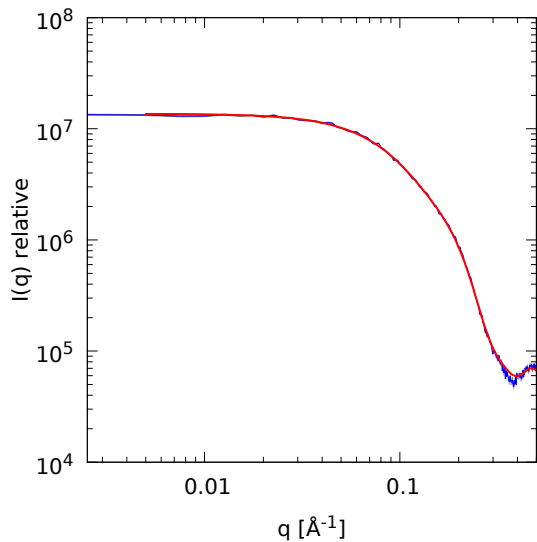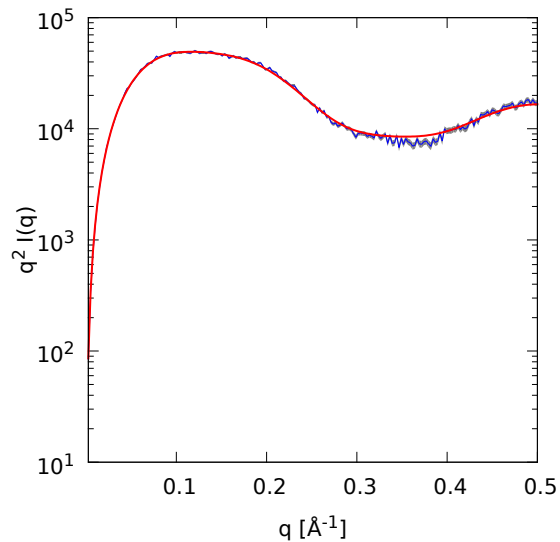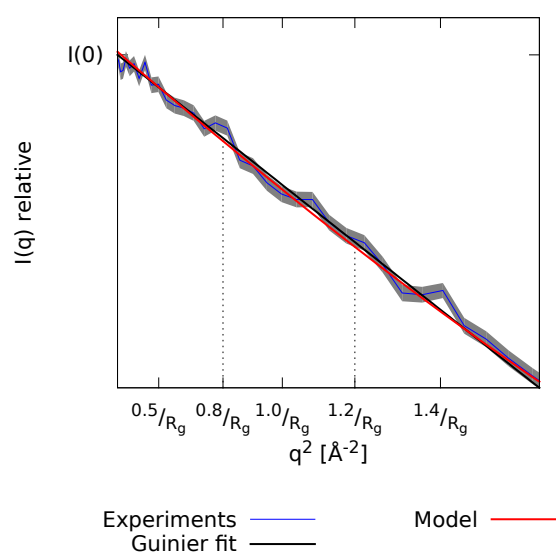

Supplement: File S6 — Benchmark data. Simulated SAXS data, fit plots, RFR models, and best ROSETTA decoys (by ) for 10 benchmark targets. Within the archive, simulated SAXS data are named in benchmark_models/*.dat, fit plots are named benchmark_models/*_fit.pdf, RFR models are named benchmark_models/*model.pdb, and top ROSETTA decoys are named rosetta_decoys/*.pdb. (ZIP) [file pone.0078007.s006.zip › benchmark_models/1XJR_fit.pdf]

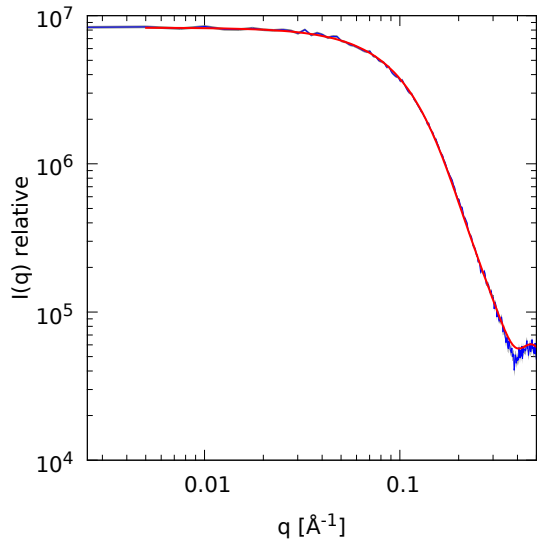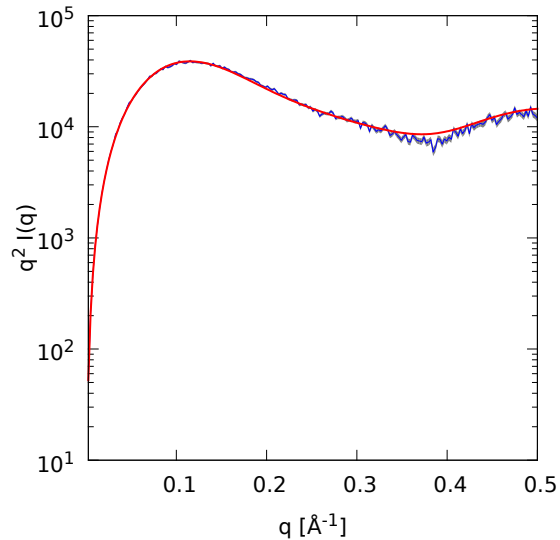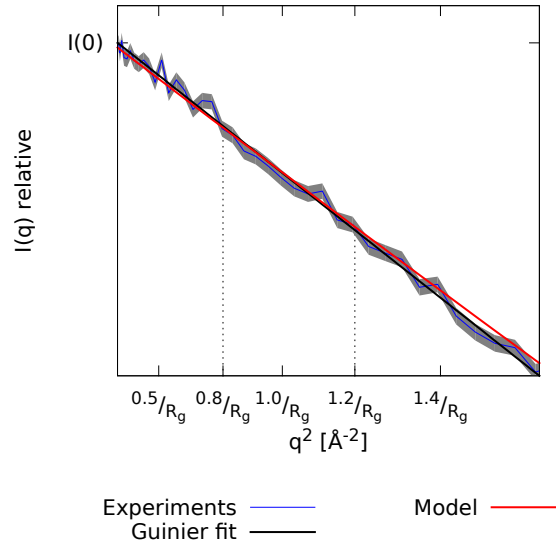

Supplement: File S6 — Benchmark data. Simulated SAXS data, fit plots, RFR models, and best ROSETTA decoys (by ) for 10 benchmark targets. Within the archive, simulated SAXS data are named in benchmark_models/*.dat, fit plots are named benchmark_models/*_fit.pdf, RFR models are named benchmark_models/*model.pdb, and top ROSETTA decoys are named rosetta_decoys/*.pdb. (ZIP) [file pone.0078007.s006.zip › benchmark_models/1DDY_fit.pdf]

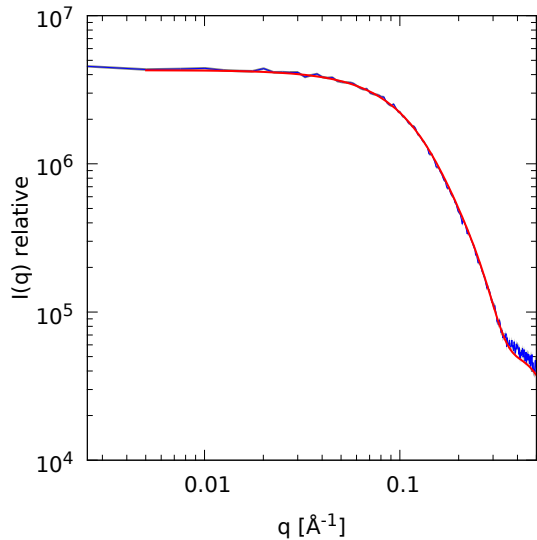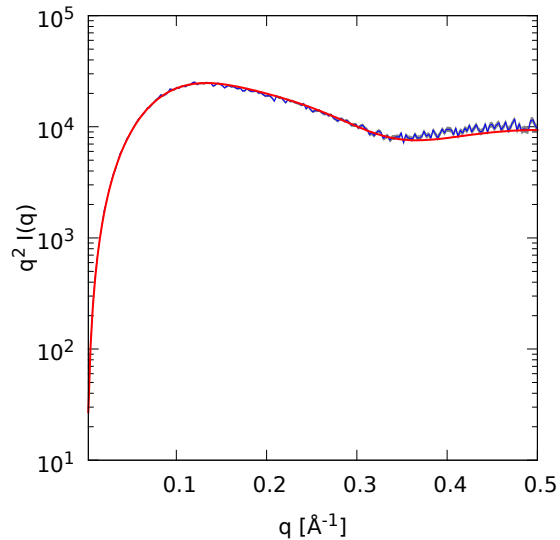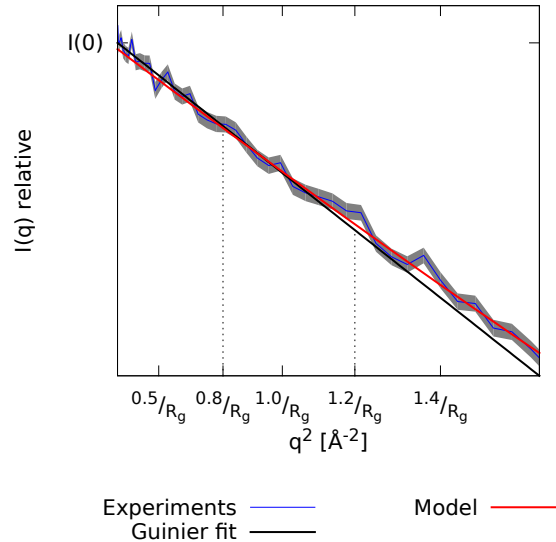

Supplement: File S6 — Benchmark data. Simulated SAXS data, fit plots, RFR models, and best ROSETTA decoys (by ) for 10 benchmark targets. Within the archive, simulated SAXS data are named in benchmark_models/*.dat, fit plots are named benchmark_models/*_fit.pdf, RFR models are named benchmark_models/*model.pdb, and top ROSETTA decoys are named rosetta_decoys/*.pdb. (ZIP) [file pone.0078007.s006.zip › benchmark_models/1Q9A_fit.pdf]

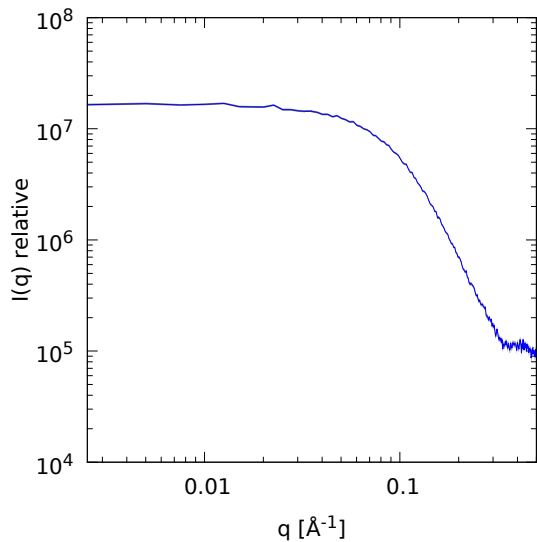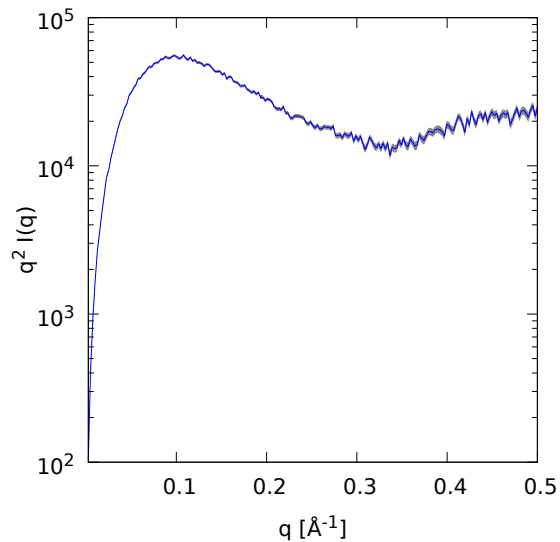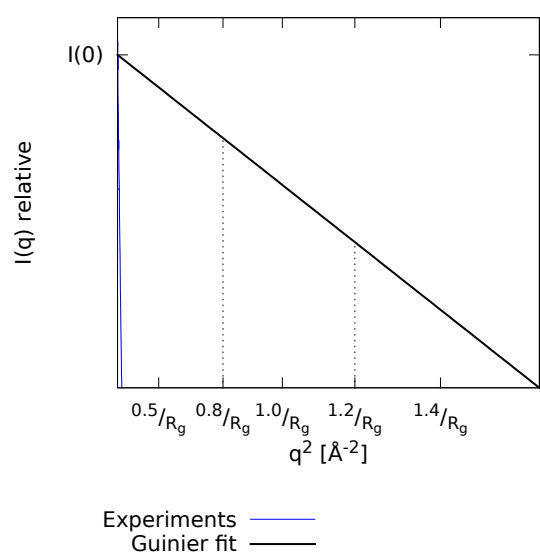

Supplement: File S6 — Benchmark data. Simulated SAXS data, fit plots, RFR models, and best ROSETTA decoys (by ) for 10 benchmark targets. Within the archive, simulated SAXS data are named in benchmark_models/*.dat, fit plots are named benchmark_models/*_fit.pdf, RFR models are named benchmark_models/*model.pdb, and top ROSETTA decoys are named rosetta_decoys/*.pdb. (ZIP) [file pone.0078007.s006.zip › benchmark_models/3E5C_fit.pdf]

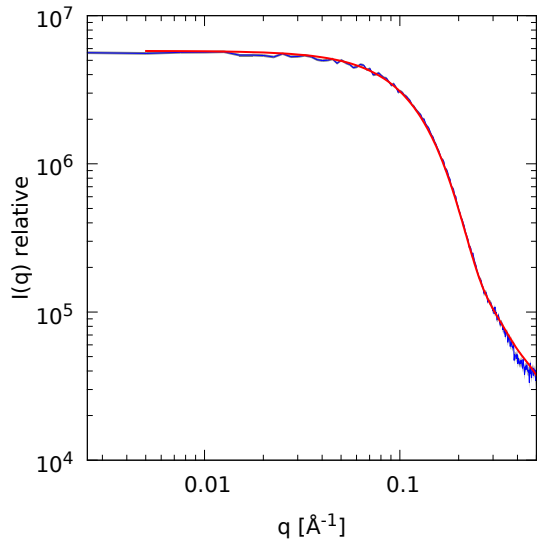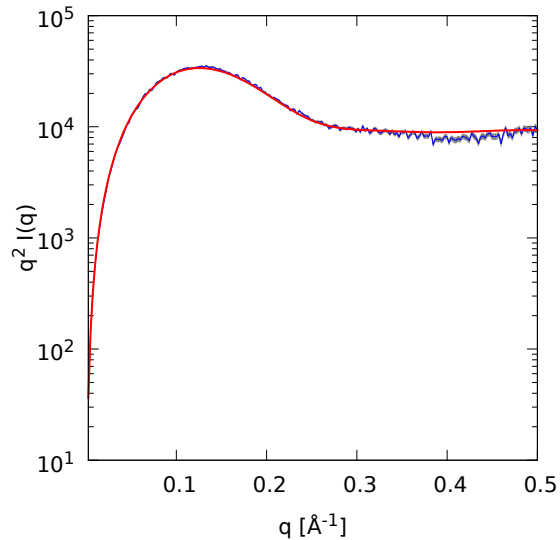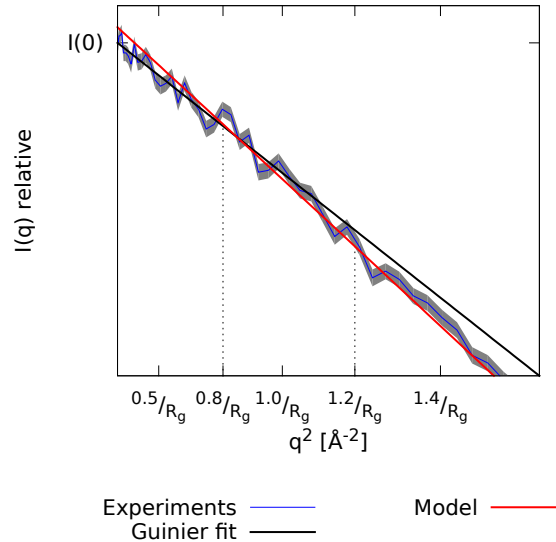

Supplement: File S6 — Benchmark data. Simulated SAXS data, fit plots, RFR models, and best ROSETTA decoys (by ) for 10 benchmark targets. Within the archive, simulated SAXS data are named in benchmark_models/*.dat, fit plots are named benchmark_models/*_fit.pdf, RFR models are named benchmark_models/*model.pdb, and top ROSETTA decoys are named rosetta_decoys/*.pdb. (ZIP) [file pone.0078007.s006.zip › benchmark_models/1L2X_fit.pdf]

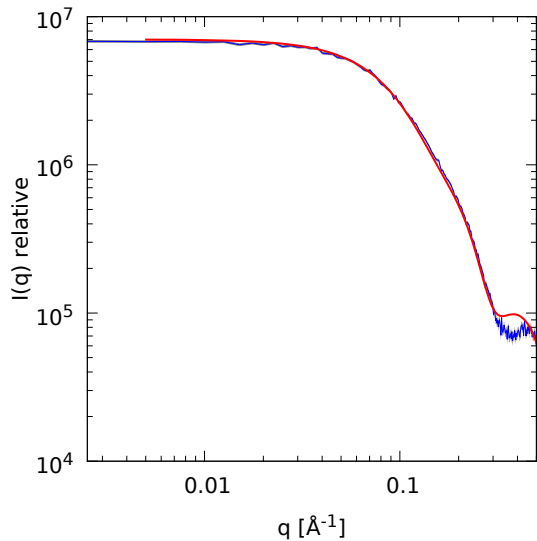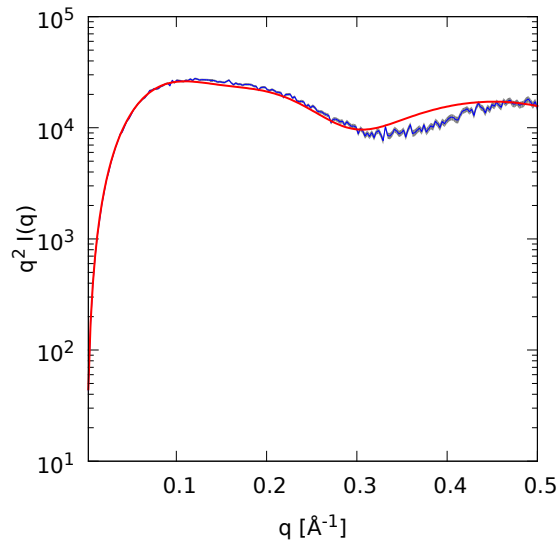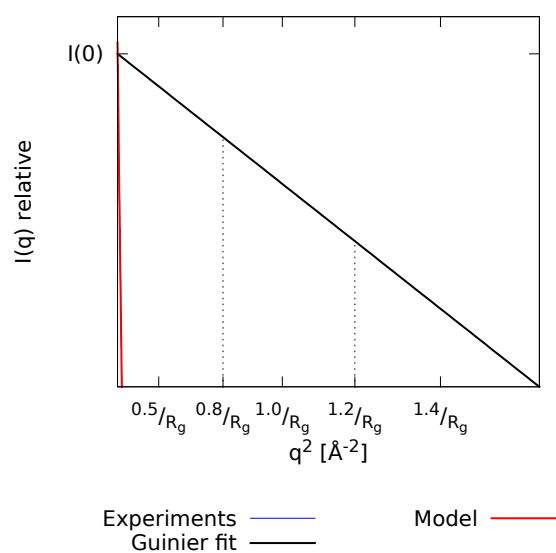

Supplement: File S6 — Benchmark data. Simulated SAXS data, fit plots, RFR models, and best ROSETTA decoys (by ) for 10 benchmark targets. Within the archive, simulated SAXS data are named in benchmark_models/*.dat, fit plots are named benchmark_models/*_fit.pdf, RFR models are named benchmark_models/*model.pdb, and top ROSETTA decoys are named rosetta_decoys/*.pdb. (ZIP) [file pone.0078007.s006.zip › benchmark_models/2L3E_fit.pdf]
